# Predictors of length of stay in psychiatry: analyses of electronic medical records

**DOI:** 10.1186/s12888-015-0623-6

**Published:** 2015-10-07

**Authors:** Jan Wolff, Paul McCrone, Anita Patel, Klaus Kaier, Claus Normann

**Affiliations:** 1 Institute of Psychiatry, Psychology & Neuroscience, King’s Health Economics, King’s College London, De Crespigny Park, SE5 8AF London, United Kingdom; 2 Department for Management and Controlling, Medical Centre-University of Freiburg, Hugstetter Strasse 49, 79106 Freiburg, Germany; 3 Barts and the London School of Medicine and Dentistry, Centre for Primary Care and Public Health, Queen Mary University of London, 58 Turner Street, E12AB London, United Kingdom; 4 Institute of Medical Biometry, Medical Centre-University of Freiburg, Hugstetter Strasse 49, 79106 Freiburg, Germany; 5 Department of Psychiatry and Psychotherapy, Medical Centre- University of Freiburg, Hauptstrasse 5, 79106 Freiburg, Germany

**Keywords:** Mental health, Hospitals, length of stay, Costs and cost analysis, Prospective payment systems

## Abstract

**Background:**

Length of stay is a straightforward measure of hospital costs and retrospective data are widely available. However, a prospective idea of a patient’s length of stay would be required to predetermine hospital reimbursement per case based on patient classifications. The aim of this study was to analyse the predictive power of patient characteristics in terms of length of stay in a psychiatric hospital setting. A further aim was to use patient characteristics to predict episodes with extreme length of stay.

**Methods:**

The study included all inpatient episodes admitted in 2013 to a psychiatric hospital. Zero-truncated negative binomial regression was carried out to predict length of stay. Penalized maximum likelihood logistic regressions were carried out to predict episodes experiencing extreme length of stay. Independent variables were chosen on the basis of prior research and model fit was cross-validated.

**Results:**

A total of 738 inpatient episodes were included. Seven patient characteristics showed significant effects on length of stay. The strongest increasing effects were found in the presence of affective disorders as main diagnosis, followed by severity of disease and chronicity of disease. The strongest decreasing effects were found in danger to others, followed by the presence of substance-related disorders as main diagnosis, the daily requirement of somatic care and male gender. The squared correlation between out-of-sample predictions and observed values was 0.14. The root-mean-square-error was 40 days.

**Conclusion:**

Prospectively defining reimbursement per case might not be feasible in mental health because length of stay cannot be predicted by patient characteristics. Per diem systems should be used.

## Background

Understanding differences in resource use between groups of patients is vital for the efficient organisation of health care [1]. Respective data are required by health care providers for managerial decision-making, such as determining appropriate staffing levels and controlling of budgets [2, 3]. Moreover, they inform policy makers considered with the efficient allocation of funds on a system level, for instance by the design of reimbursement schemes [4].

New reimbursement schemes are being implemented in the UK and in Germany based on patient classification systems [5, 6]. The common aim is to adjust reimbursement rates to assumed differences in hospital costs between groups of patients. Length of stay is a straightforward measure of hospital costs per case. Its documentation is uncomplicated and retrospective data are widely available. Therefore, it is a common unit of resource use for the evaluation of health care policies and interventions [7]. However, a prospective idea of a patient’s length of stay would be required to define groups of homogenous resource use and corresponding reimbursement rates per case based on patient classification systems.

Tulloch et al. systematically reviewed studies from the last 30 years in 2011. They have found a set of patient characteristics of rather consistent effects but confirmed only modest explanation of patients’ length of stay [8]. Moreover, included studies were rather old and the review was restricted to studies carried out in the USA. Therefore, the transferability of results to current clinical practices outside the USA, for instance to European hospital settings, remains unclear. More recent primary studies of predictors of length of stay have been carried out restricted to specific settings and patient groups (see for instance [9, 10]) but did not aim to provide a comprehensive picture of all current evidence.

The aim of this study was to analyse the predictive power of patient characteristics in terms of length of stay in a European hospital setting. A further aim was to use patient characteristics to predict episodes with extreme length of stay.

## Methods

The study included all inpatient episodes admitted in 2013 to the psychiatric hospital of the Medical Centre at the University of Freiburg, Germany. All episodes were discharged by 1^st^ of January 2015. The hospital has eight wards with a total of 120 beds. It provides care to about 1000 inpatient episodes per year.

The association of patient- and service-related characteristics with length of stay was analysed using zero-truncated negative binomial regressions to account for the identified overdispersion in count data. Penalized maximum likelihood logistic regressions were carried out to estimate probabilities of episodes to experience a very long stay and a very short stay, respectively.

Length of stay was derived from the patient administration database. Days of admission and discharge were counted as full days. Days of absence from the hospital within a patient’s stay were subtracted from total length of stay if absence was at least a full calendar day. Inpatient days associated with readmission after formal discharge and the days of the index episode were added to calculate the complete length of stay if time away was no more than 2 weeks to adjust for immature discharges of episodes that eventually required and received longer stays (*n* = 73).

Patient characteristics were documented at admission by physicians and nurses and stored in the electronic medical records. Records were regularly checked for completeness and plausibility by trained staff and demands for documentation related to missing or implausible values were sent out timely. Listwise deletion was carried out if data were eventually missing (3 %), since negative effects on validity of results were unlikely ([11], p.433). The data generating process and its use for scientific purpose was conducted in full compliance with the ethics committee of the Albert-Ludwigs-University of Freiburg.

Zero-truncated negative binomial regressions were carried out to predict length of stay, accounting for the identified overdispersion in count data [12]. Multiple episodes of the same patient were clustered to adjust standard errors. Independent variables were checked for non-linear influence on length of stay using locally weighted regressions and partial residual plots. Interactions between independent variables were investigated. Furthermore, outlier observations with high influence on coefficients were identified and these episodes were checked in detail for plausibility.

Independent variables were chosen on the basis of prior research, i.e. all variables that were both reported in the most recent comprehensive systematic review [8] and available from the electronic medical records, in order to contextualise results on theoretical grounds and avoid spurious findings of explorative nature.

Split sample cross-validation was carried out. The first half of episodes by admission date was used as estimation sample. The other half was used as validation sample. Measures of model fit were (a) the squared correlation between the observed length of stay and the predicted length of stay (R^2^) and (b) the square-root of the mean squared differences between the observed length of stay and the predictions (RMSE) [13].

Penalized maximum likelihood logistic regressions were carried out to estimate probabilities of episodes to experience a very long stay and a very short stay, respectively [14, 15]. A heuristic approach was purposefully chosen to define long and short-stay episodes, meaning a length of stay in the fourth and the first quartile of the sample, respectively. The predictive power was assessed using receiver operating characteristic curves (ROC).

## Results

A total of 738 inpatient episodes fulfilled the inclusion criteria. Table 1 shows patient and service characteristics. The mean length of stay was 58 days. Its distribution was right skewed with a mean higher than the median and a positive coefficient of skewness of 1.27. The largest diagnostic group was affective disorders, representing 46 % of all included episodes.

**Table 1:** Basic patient and service characteristics

Number of patients	671
Number of episodes	738
Length of stay, mean (sd)	58 (41)
Minimum	1
25th percentile	23
Median	51
75th percentile	80
Maximum	252
Coefficient of skewness	1.27
Age, mean (sd)	44 (17)
Female, percentage	57
ICD-10 main diagnosis, percentage	
F1 Substance	15
F2 Psychotic	14
F3 Affective	46
F4 Neurotic	10
F6 Personality	6
Others	9

*ICD* = International classification of diseases, *sd* = standard deviation

**Fig. 1:**
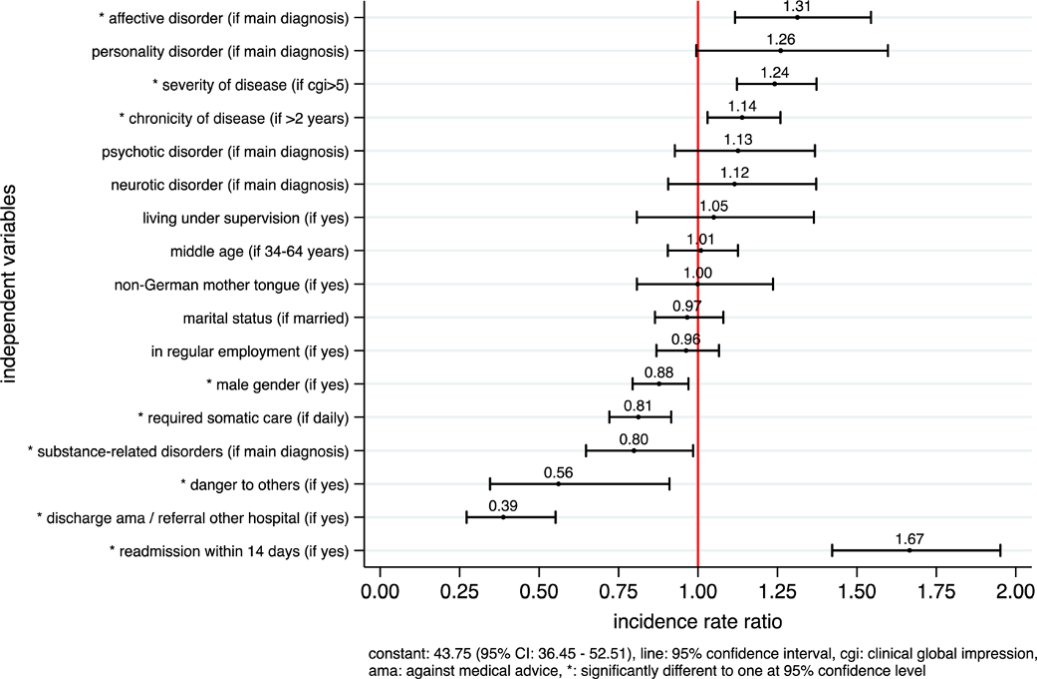
Exponentiated coefficients of zero-truncated negative binomial regression

Figure 1 shows the exponentiated coefficients of independent variables derived from the zero-truncated negative binomial regression. The strongest significantly positive effect in patient-related variables was found for the presence of affective disorders as main diagnosis. Its exponentiated coefficient of 1.31, i.e. the incidence rate ratio, represents a 31 % increase in length of stay associated with a main diagnosis of affective disorders, holding all other variables constant. Further significantly positive association of patient-variables with length of stay were found in severity of disease and chronicity of disease. The service-variable readmission after formal discharge was associated with an increase in length of stay of 67 %.

The strongest significantly negative effects of patient-variables were found in danger to others, followed by the presence of substance-related disorders as main diagnosis, the daily requirement of somatic care and male gender. The service-related variables discharge against medical advices/referral to another hospital and readmissions had significant effects on length of stay and were included in order to control for premature discharges.

Table 2 compares the model fit in the complete sample with the cross-validated fit in the estimation and validation samples. The best model fit was found in the first half of episodes admitted, i.e. in the estimation sample. The fit in the complete sample was lower than in the estimation sample, which will be addressed in more detail in the discussion. The out-of-sample predictions yielded an R^2^-statistic of 14 % and a RMSE of 40 days.

**Table 2:** Model fit in complete sample and in cross-validation

	R^2^ (%)	Root-Mean-Square-Error
Complete sample	21.95	36.08
*n* = 738	(16.26–27.95)	(33.43–39.43)
Estimation sample (in-sample)	27.80	32.89
*n* = 365	(20.95–35.52)	(29.94–38.03)
Validation sample (out-of-sample)	13.59	40.14
*n* = 373	(7.04–21.6)	(36.05–45.55)

In parantheses: 95 % confidence intervals, bootstrapped, 2000 repetitions, bias-corrected and accelerated

**Fig. 2:**
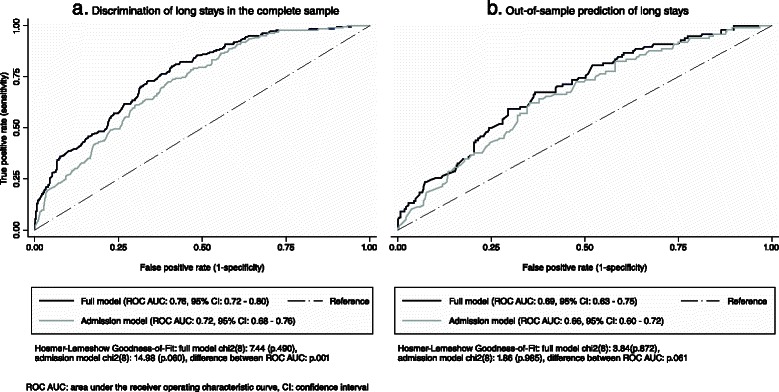
Receiver operating characteristic curves of episodes with long stays. **a** Discrimination of long stays in the complete sample. **b** Out-of-sample prediction of long stays

**Fig. 3:**
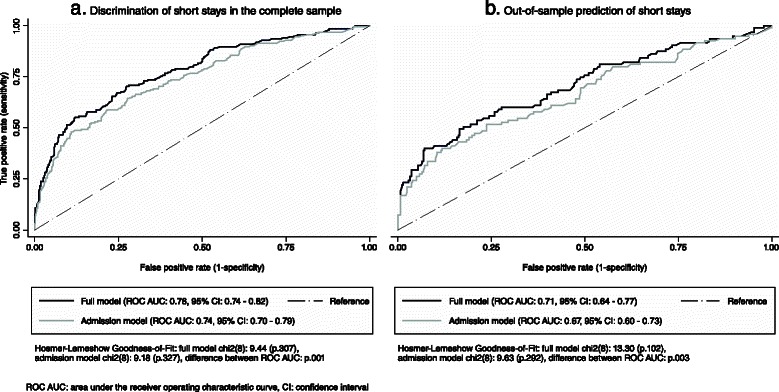
Receiver operating characteristic curves of episodes with short stays. **a** Discrimination of shortstays in the complete sample. **b** Out-of-sample prediction of short stays

Figure 2a shows the ability of the independent variables for discrimination of episodes experiencing a very long stay in the complete sample. Figure 2b shows the ability for out-of-sample predictions of very long stays. A second model (admission model) was plotted for each analysis with all variables from the full model except of discharge against medical advice/referral to another hospital and readmissions, in order to model solely on the basis of variables that were documented at admission. The presented ROCs plot the percentage of episodes rightly classified as long stays against the percentage of episodes falsely classified as long stays for any given cut of point of calculated probabilities. Figure 3 illustrates the corresponding results for the prediction of very short stays.

An “acceptable” to “low” level of accuracy was found according to common standards in the complete sample and in out-of-sample prediction, respectively [16]. For instance, an area under the ROC of 0.76 represented a probability of 76 % that a random episode experiencing a very long stay will have a higher predicted probability to do so than a random episode that does not.

## Discussion

The aim of this study was to analyse the predictive power of patient characteristics in terms of length of stay. A further aim was to use patient characteristics to predict episodes with extreme length of stay. Significant effects on length of stay were found in seven patient characteristics. Affective disorder as main diagnosis, severity of disease and chronicity of disease had increasing effects on length of stay. Danger to others, substance-related disorders as main diagnosis, the daily requirement of somatic care and male gender had decreasing effects on length of stay. The cross-validated fit of the model was medium [17] (R^2^:14 %) and the RMSE in out-of-sample prediction comparably high (40 days). Furthermore, the accuracy of out-of-sample prediction of extreme length of stay was low according to common standards [16].

### Strength and weaknesses of the study

Strength of this study was the relatively large number of patient characteristics available from the electronic medical records. Moreover, these data were consistent because their completeness and plausibility was routinely checked and demands for documentation related to missing values were sent out timely. Furthermore, patient-related variables used for prediction were documented at admission. Hence, they were external factors and therefore appropriate measures for prospective payment systems.

A limitation of this study was its single site design. Therefore, results reflect the care provided at the study site and they potentially incorporate local idiosyncrasies. This raises the issue of generalizability. However, the study sample was relatively large with care provided at eight wards for 738 inpatient episodes across all main diagnostic groups. Therefore, a rather comprehensive picture was acquired. Nevertheless, it remains uncertain whether specific patient variables would lead to other effects on length of stay under different clinical circumstances and different types of patients.

### Results in relation to prior research

The direction of effects found in significant coefficients of patient characteristics was as expected and in concurrence with related findings of prior research and clinical experiences. These were affective disorders [18], severity of disease [19], chronicity of disease [20], male gender [21] and substance related disorders [22]. The direction of effects found in danger to others and daily requirement of somatic care might appear less intuitively and are discussed below.

The decreasing effects on length of stay in documented danger to others might appear in contrast to a first intuitive assumption that more dangerous patients should require longer stays. However, Warnke et al. [23] found patients with increased hostility to experience significantly shorter stays. Boot et al. [24] found aggression to be a significant predictor of short stays. Moreover, Lansing et al. [25] found no differences in length of stay between patients that are dangerous to others and patients that are not. A possible explanation for shorter instead of longer length of stay associated with documented danger to others found by the presented study is concurrent lack of treatment compliance, which could have led to earlier discharges. This could have been robust to controlling for discharge against medical advice, since lack of compliance might have resulted in consented discharges. Furthermore, since the condition of dangerousness was documented at admission, stabilisation of acute crises during the first days of stay might have allowed early discharges.

Furthermore, the presented study found the daily requirement of somatic care to be associated with shorter length of stay. This is in contrast to most of prior research and counterintuitive. For instance, Lyketsos et al. [26] found that somatic illness documented as ‘a focus of care’ was related to longer length of stay in psychiatric inpatients. Sloan et al. [27] and Schubert et al. [28] found somatic comorbidities associated with longer length of stay in depressed patients. A probably relevant difference between these studies and the presented study is that the former were carried out in the USA and overall length of stay was substantially shorter. For instance, the mean length of stay in Lyketsos et al. [26] ranged between 7.5 days and 13.2 days and somatic illness as ‘a focus of care’ was associated with an increase of 3.2 days. While the requirement to alleviate acute somatic ailments before discharge in these studies might have prolonged relatively short stays, other factors in the context of somatic ailments might have shortened relatively long stays in the presented study, such as problems of the patient to participate in therapeutic measures. Comparable results of decreasing length of stay related to medical comorbidities was also found by Ismail et al. [10], but only for patients with dementia.

The model showed a ‘medium’ to ‘large’ fit (22 %) in the complete sample according to common standards in the social sciences [17] and in comparison to prior research findings. Of twenty studies included in a recent systematic review [8], eight studies showed better fit, ten studies showed lower fit and two studies did not report respective statistics.

Split sample cross-validation was carried out by using the one half of episodes admitted first for estimation and the other half for validation. This approach was taken instead of separating by chance in order to come closer to the concept of applying the model to external patients, who might be subject to changes in clinical circumstances [29]. As expected, the model fit was better in the estimation sample (R^2^ = 28 %) than in the validation sample (R^2^ = 14 %). Furthermore, estimating the model for the first half of admitted patients yielded a better performance than for the complete sample (R^2^ = 22 %). A potential reason for this difference might be the higher mean length of stay and variance in patients admitted at the end of the year, i.e. 64 days in quarter four compared to 55 days in quarter one to three (p.003).

A more informative but far less reported measure of predictive accuracy is the RMSE, which is a measure of the mean deviation of the modelled from the observed length of stay. The RMSE of 40 days found by the presented study in out-of sample-prediction was high with respect to a median of 56 days and an interquartile range of 59 days in the validation sample (not shown is results). However, the poor performance in prediction of individual episodes’ length of stay and concomitant strong and highly significant covariates was not a contradiction but found to be a common trait of results from predictive modelling [30].

## Conclusion

The results of this study showed that it was not possible to infer length of stay reliably from patient characteristics. Health care providers cannot rely on such data for decision-making but have to assess patients on a case-by-case basis. Policy makers considered with the efficient allocation of funds on a system level, for instance by the design of reimbursement schemes, should avoid per case systems if they want to allocate funds according to patient classification systems because homogenous groups of hospital costs per case would probably be infeasible. Instead, per diem systems should be used and adjusted to potential difference in daily resource between patient groups.

A recent systematic review has shown that there is a lack of current evidence considering differences in daily resource use between patient groups in European hospital settings [31]. A further recent study has provided unprecedented per diem unit costs of inpatient mental health care in a European setting but was mainly focused on differences between diagnostic groups and has not included many patient variables [32]. Therefore, future research should delineate patient-specific per diem resource use and relate the results to detailed patient characteristics.

## Abbreviations

AMA: Against medical advice
CI: Confidence interval
ICD: International classification of diseases
ROC: Receiver operating characteristic curve
ROC AUC: Area under the receiver operating characteristic curve
RMSE: Root-mean-square-error
R2: The squared correlation between the observed length of stay and the predicted length of stay
SD: Standard deviation
